# Social preferences correlate with cortical thickness of the orbito-frontal cortex

**DOI:** 10.1093/scan/nsab074

**Published:** 2021-07-09

**Authors:** Andrea Fariña, Michael Rojek-Giffin, Jörg Gross, Carsten K W De Dreu

**Affiliations:** Institute for Psychology, Leiden University, Leiden 2300 RB, The Netherlands; Leiden Institute for Brain and Cognition, Leiden University, Leiden 2300 RB, The Netherlands; Institute for Psychology, Leiden University, Leiden 2300 RB, The Netherlands; Leiden Institute for Brain and Cognition, Leiden University, Leiden 2300 RB, The Netherlands; Institute for Psychology, Leiden University, Leiden 2300 RB, The Netherlands; Leiden Institute for Brain and Cognition, Leiden University, Leiden 2300 RB, The Netherlands; Institute for Psychology, Leiden University, Leiden 2300 RB, The Netherlands; Leiden Institute for Brain and Cognition, Leiden University, Leiden 2300 RB, The Netherlands; Center for Research in Experimental Economics and Political Decision Making, University of Amsterdam, Amsterdam, 1018 WB, The Netherlands

**Keywords:** brain anatomy, social value orientation, decision-making

## Abstract

Humans differ in their preferences for personal rewards, fairness and others’ welfare. Such social preferences predict trust, public goods provision and mutual gains bargaining and have been linked to neural activity in regions involved in reward computation, cognitive control and perspective-taking. Although shaped by culture, social preferences are relatively stable across time, raising the question whether differences in brain anatomy predict social preferences and their key components—concern for personal outcomes and concern for others’ outcomes. Here, we examine this possibility by linking social preferences measured with incentivized economic games to 74 cortical parcels in 194 healthy humans. Neither concerns for personal outcomes nor concerns for the outcomes of others in isolation were related to anatomical differences. However, fitting earlier findings, social preferences positively scaled with cortical thickness in the left olfactory sulcus, a structure in the orbital frontal cortex previously shown to be involved in value-based decision-making. Consistent with work showing that heavier usage corresponds to larger brain volume, findings suggest that pro-social preferences relate to cortical thickness in the left olfactory sulcus because of heavier reliance on the orbital frontal cortex during social decision-making.

## Introduction

As group-living animals, many of the decisions humans make not only affect their own outcomes but those of others around them as well. When maximizing personal gains comes at a cost to others, humans need to trade off their preference for personal reward against preferences for fairness and others’ welfare (Fehr and Camerer, 2007; Van Dijk and De Dreu, 2021). How individuals make this trade-off defines their social preferences. Humans differ in their concern for fairness and others’ welfare, and such differences in social preferences explain decision-making in a variety of situations, including extending and reciprocating trust (Ashraf *et al.*, 2006; Kanagaretnam *et al.*, 2009), providing for public goods (Balliet *et al.*, 2009) and seeking a mutually beneficial outcome in negotiations (De Dreu *et al.*, 2000). Brain imaging studies have linked social preferences to neural activity in the dorsolateral prefrontal cortex associated with monitoring and executive control (Baumgartner *et al.*, 2011), the amygdala and insular cortex associated with the regulation of emotion (Haruno *et al.*, 2014; Liu *et al.*, 2019) and the temporoparietal junction associated with empathy and perspective-taking (namely, Theory of Mind; Emonds *et al.*, 2014; for recent reviews, see De Dreu, Gross, Fariña & Ma, 2020; Hung *et al.*, 2020; Amodio and Cikara, 2021).

Being pivotal to living and working in groups, social preferences are shaped by culture and socialization on the one hand (Van Lange, 1999) and evolutionary selection pressures on the other (Darwin, 1859; Bowles, 2009). Akin to personality traits, social preferences are relatively stable across time and operate in addition to situational pressures (Van Lange, 1999; Murphy and Ackerman, 2014; De Dreu and Gross, 2019). Personality traits related to social preferences, such as agreeableness, have been shown to be related to differences in brain anatomy (Riccelli *et al.*, 2017). Along similar lines, Churchwell and Yurgelun-Todd (2013) found a positive relationship between insula thickness and impulsivity, while Muhlert and Lawrence (2015) identified that rash impulsivity correlated with lower volume of the ventral striatum.

**Fig. 1.**
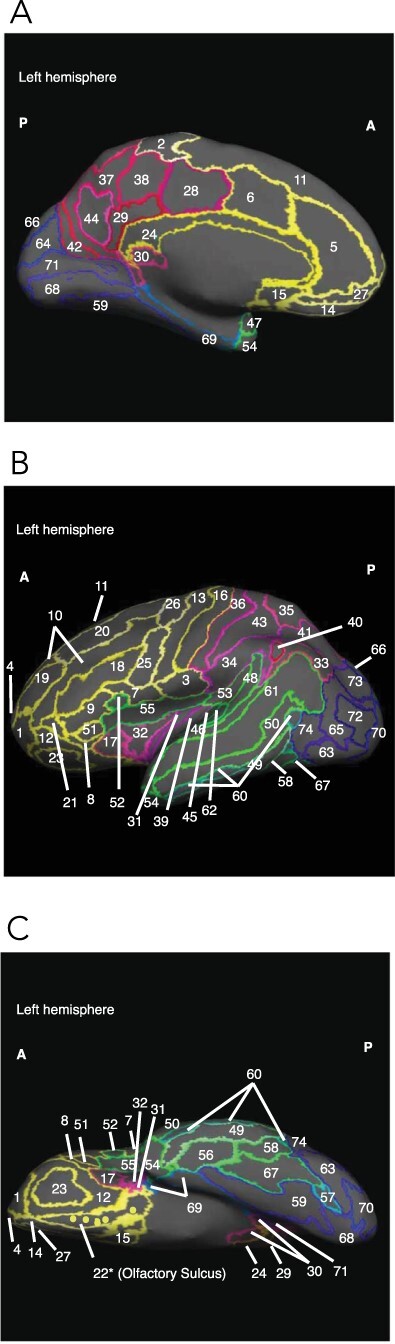
Brain regions by lobe. (A) Medial view of the left hemisphere; (B) lateral view of the left hemisphere; (C) inferior view of the left hemisphere. Yellow hues indicate frontal regions, green indicates the temporal lobe, pink is parietal and blue is occipital. Numbers indicate different parcels.

Here, we examine the possibility that social preferences are likewise associated with differences in brain structure and anatomy. Indeed, previous research has shown that cortical thickness of the dorsolateral prefrontal cortex predicts strategic choices in economic games (Yamagishi *et al.*, 2016) and that gray matter volume in the right temporoparietal junction varies with individual differences in altruistic giving (Morishima *et al.*, 2012). Currently lacking, however, is a statistically powerful and comprehensive whole-brain analysis on the relationship between general social preferences and anatomical differences. Here we fill this gap. We measured social preferences using a standardized and incentivized decision task in a large sample of healthy male and female volunteers. Earlier work using this task showed that social preferences reliably predict HEXACO Honesty-Humility and Big Five Agreeableness scores, cooperation in economic games, as well as charitable donations and involvement in volunteering (Pletzer *et al.*, 2018; Van Dijk and De Dreu, 2021). We investigate how pro-social preferences, and the underlying concern for personal rewards and concern for others’ rewards, relate to cortical thickness of 74 distinct bilateral brain areas with identifiable functionalities for human cognition and behavior. Figure 1 shows a graphical display of these anatomical structures for the left hemisphere.

To guide our analyses, we reviewed findings from functional neuroimaging studies linking brain regions to social decision-making. Social decision-making has been argued to rely on the capacity to form expectations of others’ cooperative behavior (Declerck and Bogaert, 2008; Emonds *et al.*, 2011), on the cognitive control of both selfish and pro-social impulses (Baumgartner *et al.*, 2011; Rand *et al.*, 2013) and on the ability to perform broad utility-based calculations (Fehr and Schmidt, 2006). Table 1 shows, for each anatomical parcel in Figure 1, its mapping on functional regions and neural networks. We identified three (partly overlapping and interacting) neural networks with well-documented functionalities for social preferences and concern for self and others in particular: the Cognitive Control Network that includes the dorsolateral prefrontal cortex, the anterior cingulate and the orbitofrontal cortex (OFC) (Duncan and Owen, 2000; Aron, Robins, & Poldrack, 2004); the Default Mode Network that includes the (ventro)medial prefrontal cortex, the posterior cingulate cortex (precuneus), the temporoparietal junction and the anterior temporal pole and cortex (Buckner *et al.*, 2008; Blakemore, 2008; Bressler and Menon, 2010; Wittmann *et al.*, 2018); and the Theory of Mind Network that includes the medial prefrontal cortex, the superior temporal sulcus, the precuneus, the temporoparietal junction, the anterior cingulate cortex and the inferior frontal gyrus (Gallagher and Frith, 2003; Spreng *et al.*, 2009; Decety, 2010; Schurz *et al.*, 2014). Whether and to what extent anatomical features of these or other parcels are associated with social preferences remain largely unknown and are examined here in terms of cortical thickness.

**Table 1.** Anatomical parcels by lobe according to the Destrieux Atlas (ID, see Figure 1), with associated functional regions and neural networks involved in empathy, (cognitive) control and reward processing

ID	Anatomical parcel	Functional regions (networks)	Empathy	(Cognitive) control	Reward processing
**Frontal lobe**					
1	Frontomarginal gyrus and sulcus	VMPFC; OFC (DMN; ToM)	Moll *et al.* (2002); Lewis *et al.* (2011)	Koechlin and Hyafil (2007)	Hare *et al.* (2009); Levy and Glimcher (2012)
2	Paracentral gyrus and sulcus	SMA	Fan *et al.* (2011)	–	–
3	Subcentral gyrus and sulcus	SMA	Adolphs *et al.* (2000); Molenberghs *et al.* (2012)	–	–
4	Transverse frontopolar gyri	VMPFC; OFC (CNN, DMN; ToM)	Shamay-Tsoory *et al.* (2009); Lewis *et al.* (2011)	Koechlin and Hyafil (2007); Boorman *et al.* (2009)	Hare *et al.* (2009); Levy and Glimcher (2012)
5	Anterior cingulate gyrus and sulcus	ToM	Van Overwalle and Baetens (2009); Schurz *et al.* (2014)	Botvinick *et al.* (2001); Kerns *et al.* (2004)	Kennerley *et al.* (2006)
6	Mid anterior cingulate gyrus and sulcus	ToM	Lamm *et al.* (2011)	Shackman *et al.* (2011)	Kennerley *et al.* (2006)
7	Pars opercularis	IFG (ToM)	Schurz *et al.* (2014)	Derrfuss *et al.* (2005)	–
8	Pars orbitalis	IFG (ToM)	Schurz *et al.* (2014)	Derrfuss *et al.* (2005)	–
9	Pars triangularis	IFG (ToM)	Schurz *et al.* (2014)	Derrfuss *et al.* (2005)	–
10	Middle frontal gyrus	DLPFC (CCN)	–	Buchsbaum *et al.* (2005); Hare *et al.* (2009)	–
11	Superior frontal gyrus	SMA	Farrow *et al.* (2001); Fan *et al.* (2011)	Bonini *et al.* (2014)	–
12	Orbital gyri	OFC (CCN; ToM)	Abu-Akel and Shamay-Tsoory (2011)	–	Levy and Glimcher (2012)
13	Precentral gyrus	M1	–	–	–
14	Rectus gyrus	OFC (CCN; ToM)	Abu-Akel and Shamay-Tsoory (2011)	–	Levy and Glimcher (2012)
15	Subcallosal gyrus		–	–	–
16	Central sulcus	M1 and S1 (ToM)	–	–	–
17	Anterior circular sulcus		Lamm *et al.* (2011)	McKenna *et al.* (2017)	Liu *et al.* (2011)
18	Inferior frontal sulcus	OFC (CCN; ToM)	Carrington and Bailey (2009)	Derrfuss *et al.* (2005); Pan *et al.* (2018)	Levy and Glimcher (2012)
19	Middle frontal sulcus		–	Buchsbaum *et al.* (2005); Hare *et al.* (2009)	–
20	Superior frontal sulcus	SMA	Fan *et al.* (2011)	Bonini *et al.* (2014)	–
21	Lateral orbital sulcus	OFC; VMPFC (CNN, DMN, ToM)	Moll *et al.* (2002); Lewis *et al.* (2011)	–	Hare *et al.* (2009); Levy and Glimcher (2012)
22	Olfactory sulcus	OFC (CCN, ToM)	Moll *et al.* (2002); Lewis *et al.* (2011)	–	Hare *et al.* (2009); Levy and Glimcher (2012)
23	H-shaped sulcus	OFC (CCN, ToM)	Moll *et al.* (2002); Lewis *et al.* (2011)	–	Hare *et al.* (2009); Levy and Glimcher (2012)
24	Pericallosal sulcus		–	–	–
25	Inferior precentral sulcus	SMA	Fan *et al.* (2011)	–	–
26	Superior precentral sulcus	SMA	Fan *et al.* (2011)	–	–
27	Suborbital sulcus	OFC (CCN, ToM)	Moll *et al.* (2002); Lewis *et al.* (2011)	–	Hare *et al.* (2009); Levy and Glimcher (2012)
**Parietal lobe**					
28	Mid posterior cingulate gyrus and sulcus	DMN	Schlaffke *et al.* (2015)	Kragel *et al.* (2018)	–
29	Dorsal posterior cingulate gyrus	DMN	Schlaffke *et al.* (2015)	Kragel *et al.* (2018)	–
30	Ventral posterior cingulate gyrus	DMN	Schlaffke *et al.* (2015)	Kragel *et al.* (2018)	–
31	Long insular gyri and central insular sulcus		–	–	–
32	Short insular gyrus		Fan *et al.* (2011)	–	–
33	Angular gyrus	TPJ (DMN, ToM)	Schurz *et al.* (2017)	Cabeza *et al.* (2012)	–
34	Supramarginal gyrus	TPJ (DMN, ToM)	Schurz *et al.* (2017)	Cabeza *et al.* (2012)	–
35	Superior parietal gyrus		–	McKenna *et al.* (2017)	–
36	Postcentral gyrus		Fan *et al.* (2011)	–	–
37	Precuneus		Schurz *et al.* (2014)	–	–
38	Marginal sulcus		–	–	–
39	Inferior circular sulcus		–	–	–
40	Jensen sulcus	TPJ (DMN, ToM)	Schurz *et al.* (2017)	Cabeza *et al.* (2012)	–
41	Intraparietal sulcus	TPJ (DMN, ToM)	Schurz *et al.* (2017)	McKenna *et al.* (2017)	–
42	Parieto occipital sulcus		–	–	–
43	Postcentral sulcus	S1 (ToM)	–	–	–
44	Subparietal sulcus		–	–	–
**Temporal lobe**					
45	Anterior transverse temporal gyri	AC (DMN)	–	–	–
46	Lateral superior temporal gyrus		Zahn *et al.* (2007)	–	–
47	Planum polare	AC	–	–	–
48	Planum temporale	AC	–	–	–
49	Inferior temporal gyrus		–	–	–
50	Middle temporal gyrus		Carrington and Bailey (2009)	–	–
51	Horizontal lateral fissure	S2	–	–	–
52	Vertical lateral fissure	S2	–	–	–
53	Posterior lateral fissure	S2	–	–	–
54	Temporal pole	DMN	Carrington and Bailey (2009)	McKenna *et al.* (2017)	–
55	Superior circular sulcus		–	–	–
56	Anterior collateral sulcus		–	–	–
57	Posterior collateral sulcus		–	–	–
58	Lateral occipital-temporal sulcus		–	–	–
59	Medial occipito-temporal and lingual sulci		–	–	–
60	Inferior temporal sulcus		–	–	–
61	Superior temporal sulcus		Molenberghs *et al.* (2016); Isik *et al.* (2017)	–	–
62	Transverse temp. sulcus	AC	–	–	–
**Occipital lobe**					
63	Inferior occ. gyrus and sulcus		–	–	–
64	Cuneus	V1	–	–	–
65	Middle occipital gyrus		Carrington and Bailey (2009)	–	–
66	Superior occipital gyrus		Carrington and Bailey (2009)	–	–
67	Fusiform gyrus		Carrington and Bailey (2009)	–	–
68	Lingual gyrus		Carrington and Bailey (2009)	Buchsbaum *et al.* (2005)	–
69	Parahippocampal gyrus		–	–	–
70	Occipital pole		–	–	–
71	Calcarine sulcus	V1	–	–	–
72	Lunatus sulcus	V1, V2	–	–	–
73	Superior and transversal occipital sulci		–	–	–
74	Anterior occipital sulcus		Carrington and Bailey (2009)	–	–

*Notes*: VMPFC = ventromedial prefrontal cortex; OFC = orbitofrontal cortex; IFG = inferior frontal gyrus; DLPFC = dorsolateral prefrontal cortex; M1 = motor cortex (primary); S1 = somatosensory cortex (primary); S2 = somatosensory cortex (secondary); V1 = visual cortex (primary); V2 = visual cortex (secondary); AC = auditory cortex; TPJ = temporoparietal junction; CCN = cognitive control network; DMN = default mode network; SMA = supplementary motor area.

## Materials and methods

### Participants and ethics

To acquire social preferences and structural brain images, 214 participants were tested individually and compensated 50 euros in addition to their earnings from decision-making (subjects were paid out one randomly drawn decision, on average earning €5). Sample size was based on earlier work on social value orientation (SVO) and cooperative decision-making in experimental games, typically showing that 40% of research samples can be classified as pro-social and another 40% as selfish (e.g. Van Lange, 1999). Our sample size fell in the upper range of earlier studies examining correlations between personality dimensions and brain anatomy (i.e. ranging between 50 < *N* < 500; see, e.g., Fischl and Dale, 2000; Haas *et al.*, 2015; Kitayama *et al.*, 2017; Morishima *et al.*, 2012; Riccelli *et al.*, 2017; Yamagishi *et al.* 2016). The experimental protocol received approval from the Ethics Review Board of the University of Amsterdam (ethics approval number 2015-EXT-4366). Participants provided written informed consent and received a full debriefing. The experiments involved no deception and decisions were fully incentivized.

### Brain imaging

All brain images were acquired using a Philips Achieva 3T MRI scanner and a 32-channel SENSE head coil at the University of Amsterdam. A survey scan was made for spatial planning of the subsequent scans. Following the survey scan, a 3-min structural T1-weighted scan was acquired using three-dimensional fast-field echo (repetition time = 82 ms, echo time = 38 ms, flip angle = 8, field-of-view = 240 × 188 mm, 220 slices acquired using single-shot ascending slice order and a voxel size of 1 × 1 × 1 mm). Cortical reconstruction and segmentation were performed with the Freesurfer image analysis suite (http://surfer.nmr.mgh.harvard.edu/). This processing included removal of non-brain tissue, automated Talairach transformation, segmentation of the subcortical white matter and deep gray matter volumetric structures, intensity normalization, tessellation of the gray matter white matter boundary and automated topology correction. Once the cortical models were complete, they were aligned to a spherical atlas which is based on individual cortical folding patterns to match cortical geometry across subjects (Fischl *et al.*, 1999), and the cerebral cortex was parcellated into 74 parcels on each hemisphere with respect to gyral and sulcal structures (Destrieux *et al.*, 2010). Cortical thickness was calculated as the closest distance from the gray/white boundary to the gray/CSF boundary at each vertex on the tessellated surface (Fischl and Dale, 2000). These outputs were then visually inspected to ensure the quality of the images and their segmentation.

### Social preferences

Either before or after brain imaging, we assessed social preferences using the SVO Ring Measure (Liebrand, 1984; Liebrand and McClintock, 1988; Van Lange, 1999). The Ring Measure has strong convergent validity with related measures of social preferences, acceptable test–retest reliability with time gaps up to 2 months (Murphy and Ackerman, 2014), and predicts trust and trustworthiness (Kanagaretnam *et al.*, 2009), public good provision (Liebrand, 1984; Pletzer *et al.*, 2018), and fairness and conciliatory behavior in bargaining (De Dreu and Van Lange, 1995). Thus, SVO measures a person’s chronic tendency to cooperate with others, as opposed to more transient measures of other-regarding preferences that may change conditional on who they are interacting with and the intentions of those they are interacting with (Fehr and Schmidt, 2006; Van Dijk and De Dreu, 2021).

The Ring Measure requires subjects to make 24 incentivized choices between pairs of own-other monetary outcomes, forcing participants to systematically trade their own economic welfare with the welfare of another person (Supplementary Table S1). Each pair represents an outcome on a circle with radius €15 and origin €15 for the self and €15 for an anonymous other (Supplementary Figure S1). Each choice involves two adjacent pairs on the circle. For instance, one question confronts participants with a choice between €28 for him/herself and €22.50 for an anonymous partner (the other) or €29.50 for the self and €18.90 for the other. The latter option increases the payoff for the participant at the expense of the other person, while the first option is more pro-social and increases the payoff of the other person at the expense of the payoff for oneself.

**Fig. 2.**
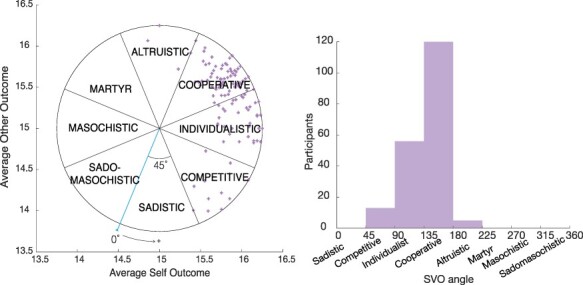
Social value orientation (SVO) categories by degree. Left: each cross represents one participant. A SVO degree of 0 corresponds to a perfectly sadistic orientation (sacrificing own outcome to reduce the outcome of the other person). As SVO degree increases, so does a person’s willingness to trade money for the self in return for money for the other. Right: histogram of participants by SVO category.

To obtain a measure of a participant’s SVO, the average amount allotted to the self is divided by the average amount allotted to the other. This ratio is then converted into an angle measurement, by taking its inverse tangent. Traditionally, the angle is used to classify subjects into distinct SVO categories (Figure 2; Supplementary Table S1 and Figure S1). Here we used the angle as a continuous measure of pro-sociality and transformed participants’ degree measures such that an angle measurement of 0° corresponds to a decision pattern that sacrifices own payoff to hurt the other person (traditionally labeled ‘sadistic’, a pattern that is very uncommon and that none of our subjects exhibited). Through this transformation, we achieved a positive correlation between the degree of the SVO angle and the participant’s willingness to trade money for self in return for money for the other person.

Next to its angle, for each participant, the SVO measure allows to calculate a vector length reflecting the consistency in allocation choices: the shorter the vector, the more inconsistent the participant (Murphy and Ackerman, 2014). A perfectly consistent participant has a vector length of 1.25. Twenty participants had a vector length shorter than 0.625 (50% of this maximum vector), indicating non-consistent random behavior across trials. This prohibits reliable inference of social preferences. We excluded these 20 participants from the main analyses, leaving a total sample of *N* = 194 with complete data (mean age = 24.15, s.d. = 1.90, 105 females). Including these 20 participants reduced effect sizes but nevertheless permitted the same conclusions. For six participants, age and gender data were missing and replaced by the average age and randomly assigned gender (omitting these subjects from the main analyses resulted in descriptively and statistically similar results). Neither gender nor age were significantly correlated with SVO angle (SVO_male_ = 137.40, SE = 0.34; SVO_female_ = 137.35, SE = 0.23; *b* = −0.05, *P* = 0.98; *r*(age, SVO) = −0.092, *P* = 0.20).

The SVO angle is a composite of each participant’s weight to self (*W*_self_) and weight to other (*W*_other_). *W*_self_ is defined as the difference of money allocated to oneself and the median amount of total money to be gained for the self (€360), divided by the maximal difference between the self and the other’s outcomes (€60). This normalizes the result such that a median score corresponds to a value of zero. Accordingly, a subject who allocates the maximum (minimum) amount of money to oneself will have a score of 0.5 (−0.5) for *W*_self_. Similarly, *W*_other_ is defined as the difference of money allocated to the other and the median amount of total money to be gained for the other (€360), divided by the maximal difference between the self and the other’s outcomes (€60). A subject who allocates the maximum (minimum) amount of money to the other will have a score of 0.5 (−0.5) for *W*_other_. These weights are in line with the underlying psychological motivations for the categories shown in Figure 2 (e.g. if a person falls within the ‘competitive’ category, it is inferred that she/he tries to maximize the positive difference between the payoff for oneself and the payoff for the other) and described in Murphy and Ackerman (2014; Supplementary Table S1). To illustrate, if a participant answered all 24 Ring Measure questions in such a way that they allocated €380.40 to themselves and €370.90 to the other, they would have a *W*_self_ = (380.40 − 360)/60 = 0.34 and a *W*_other_ = (370.90 − 360)/60 = 0.18.

### Data analytic strategy

To examine the relationship between SVO and cortical thickness, we categorized each of the cortical parcels into the four brain lobes: the frontal (27 parcels), temporal (18 parcels), parietal (17 parcels) and occipital (12 parcels) (Destrieux *et al.*, 2010; see Figure 1). The insular and the cingulate gyri and sulci (parcels numbered 5, 6, 17, 28, 29, 31, 32, 39 and 55 in Figure 1) were classified into one of these four lobes based on proximity. For each cortical parcel, we performed regressions of the type:

(1) \begin{align*} \textrm{Cortical thickness(parcel)} =&\, C+\beta_{1} \textrm{(SVO)}+\beta_{2}\textrm{(age)}+\beta_{3}\textrm{(gender)}\nonumber\\&+\beta_{4}\textrm{(average thickness)}\end{align*}

where β_1_ is the standardized regression weight for SVO, β_2_ and β_3_ the standardized weights for age and gender, respectively, β_4_ the regression weight for average thickness of the corresponding left or right hemisphere and ε estimates the error variance.

To identify whether the relationship between cortical thickness and SVO varies by hemisphere, as others have found (Morishima *et al.*, 2012; Yamagishi *et al.*, 2016), regressions were computed separately for left and right hemispheres, with the average thickness of the corresponding hemisphere included as a control variable. Table A1 (Appendix) lists the observed regression weights for each parcel. Crucially, the number of tests performed increases the risk of false-positives. We maintained this risk at a Type I error at 5% through the Freedman and Lane (1983) permutation testing procedure. Permutation tests estimate statistical significance directly from the data being used, and the estimated familywise error rate has been shown to be more reliable than when using parametric methods (Winkler *et al.*, 2014; Eklund *et al.*, 2016). Thus, to test whether β_1_ was different from zero, we first computed a null distribution of β_1_ by permuting the real data while keeping the relationship between each participant’s cortical thickness and the control variables unchanged. We then used this null distribution to find the 5% threshold at which to compare our original *t*-statistics for β_1_. Specifically, for each of the four lobes, we (i) obtained *t*-statistics for the regression coefficient β_1_ for each cortical parcel; (ii) ran reduced regression models without the variable of interest (SVO) and saved the fitted values of cortical thickness as well as the residuals from this model; (iii) permuted the residuals from the reduced model to create a permuted fitted value of cortical thickness and (iv) estimated the full regression model using the permuted fitted values from (iii) as the dependent variable

(2) \begin{align*} \textrm{Permuted cortical thickness(parcel)} =& \,C+\beta_{1} \textrm{(SVO)}+\beta_{2}\textrm{(age)}\nonumber\\&+\beta_{3}\textrm{(gender)}\nonumber\\&+\beta_{4}\textrm{(average thickness)}\end{align*}

We saved the maximum t-statistic of all parcels within each lobe; (v) repeated steps (iii) and (iv) 5000 times; (vi) compared the original *t*-statistics from equation (1) to the distribution of 5000 maximum t-statistics of step (v) to obtain a corrected *P*-value for the regression coefficient of SVO. This procedure is equivalent to testing H0 that β_1_ = 0. Permutation results are shown in Table A1 (Appendix).

## Results

**Fig. 3.**
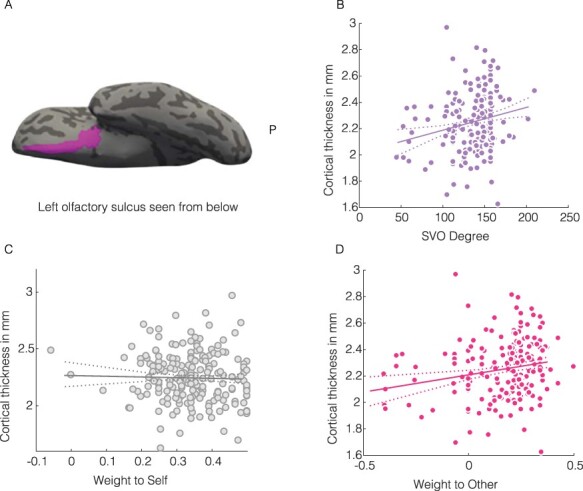
Cortical thickness of the left olfactory sulcus (OS) relates to social preferences. (A) View of left OS from below on an inflated surface. (B) SVO degree—OS pairs with best fitting linear regression, controlling for age, gender, and average thickness of the left hemisphere. (C) Cortical thickness of the left olfactory sulcus and weight to self. Solid line represents the regression coefficient of weight to self after controlling for age, gender, weight to other, and average thickness of the left hemisphere. (D) Cortical thickness of the left olfactory sulcus and weight to other. Solid line represents the regression coefficient of weight to other after controlling for age, gender, weight to self and average thickness of the left hemisphere. Dashed lines represent the 95% confidence interval. Dots represent individual participants.

Fitting previous research, we found no significant relationship between cortical thickness and SVO angle within the occipital lobe (Table A1, Appendix). Importantly, we also found no significant relations between SVO angle and regions in the temporal and parietal lobes after permutation testing - regions that earlier work associated with empathy or theory of mind (see Table A1, Appendix). At the same time, however, we did observe a robust positive relationship between SVO angle and the cortical thickness of the olfactory sulcus—an area of the OFC which is functionally involved in value-based decision-making (Table 1, Figure 3A). Stronger pro-social preferences linearly scaled with the cortical thickness of the olfactory sulcus (Figure 3B; β = 0.0017, permuted *P* = 0.03).

The SVO angle is a composite of two variables—concern for self (*W*_self_) and concern for other (*W*_other_)—that are only partially correlated within the SVO measure and may have independent (and perhaps even opposite) relations to neural regions, both functionally and structurally. Accordingly, we repeated our regression analyses substituting SVO angle by *W*_self_ and *W*_other_, using the permutation testing procedures described above for β × SVO but now with β × W_self_ and β × W_other_ as the variables of interest

(3) \begin{align*} \textrm{Cortical thickness(parcel)} =&\,C+\beta_{5}\textrm{(}W_{self}\textrm{)}+\beta_{6}\textrm{(}W_{other}\textrm{)}\nonumber\\&+\beta_{3}\textrm{(gender)}\nonumber\\&+\beta_{4}\textrm{(average thickness)}\end{align*}

After correcting for multiple comparisons using permutation testing, we found null results for the occipital, temporal, parietal lobes and *W*_self_ or *W*_other_ (Table A1, Appendix). Further qualifying results for SVO angle, we found a positive relationship between the cortical thickness of the left olfactory sulcus and *W*_other_ (Figure 3D; β = 0.26, permuted *P* = 0.06) and no such relationship with *W*_self_ (Figure 3C; β = −0.08, permuted *P* = 1). This suggests that the relationship between SVO angle and cortical thickness of the left olfactory sulcus resides in differences in the weight given to other’s outcomes and not in the weight given to own outcomes.

## Conclusions and discussion

Previous structural (Sul, et al., 2015; Nash *et al.*, 2015; Yamagishi *et al.*, 2016; Sul, Güroğlu, Crone, & Chang, 2017) and functional imaging studies (Baumgartner *et al.*, 2011; Emonds *et al.*, 2011; Balconi and Canavesio, 2014; Christov-Moore *et al.*, 2017) on cognitive control and value-based decision-making gave us reason to expect a relationship between cortical thickness in the dorsolateral prefrontal cortex (Areas 10, 19 in Figure 1A) and social preferences. Our comprehensive whole-brain analyses did not, however, reveal such linkages. Likewise, results did not show any relationship between social preferences and cortical thickness of areas in the parietal lobe that earlier work associated with Theory of Mind (i.e. the temporoparietal junction; see Saxe and Kanwisher, 2003; Van Overwalle, 2009; Molenberghs *et al.*, 2016).

These negative results aside, our comprehensive whole-brain analysis revealed a significant correlation between SVO angle and cortical thickness of the OFC (Areas 4, 12, 14, 18, 21, 22, 23 in Figures 1A and 1B). The OFC, a critical region for value-based decision-making (Rolls and Grabenhorst, 2008), has been previously linked to decisions involving social preferences (Fehr and Camerer, 2007). Kitayama and colleagues (2017) recently showed that OFC cortical volume inversely predicted interdependent self-construal—the view of the self in relation to others, while Haas and colleagues (2015) found that self-reported trust scores were positively associated with gray matter volume in the bilateral ventromedial prefrontal cortex.

Our results are in line with these findings and show that stronger prosocial preferences associate with the thickness of the left olfactory sulcus (Area 22 in Figure 1C). One possible explanation for the link between SVO angle and the OFC is that prosocial individuals take not only their own outcome into account but also those of others, rendering their decision-making process computationally more demanding than that of pro-self individuals. Indeed, previous research on SVO has shown that cooperators and competitors have, compared to individualists, longer response latencies (Liebrand and McClintock, 1988; Chen and Fischbacher, 2016) and engage in greater information search (Fiedler *et al.*, 2013) when making allocation decisions. Possibly, these more complex value computations may require greater involvement from the OFC. In line with this reasoning, research has found that sensorimotor experience leads to structural brain changes in human (Maguire *et al.*, 2000; Draganski *et al.*, 2004) and macaque (Quallo *et al.*, 2009) gray matter, especially in sensorimotor areas.^1^ Furthermore, there is some evidence that social complexity, including social network size, correlates with the size of prefrontal regions in various species, including the macaque (Sallet *et al.*, 2011; Wittmann *et al.*, 2018; also see Bickart *et al.*, 2011) and cleaner fish (Triki *et al.*, 2019). However, since the directionality of social behavior and brain anatomy is not yet fully understood, it may be the case that existing brain structure leads more readily to certain behaviors, rather than the other way around. Combined, these works suggest that extensive engagement of a particular brain region correlates with its cortical thickness, and this may explain the observed correlations between cortical thickness of the left olfactory sulcus and pro-social preferences reported here.

Our analyses covered the whole brain in a relatively large sample of healthy participants. Whereas significant correlations were found between the cortical thickness of particular brain regions and our measure of social preferences (see Table A1, Appendix), these findings did not survive rigorous permutation testing. Even in the one case that did survive permutation testing—the cortical thickness of the left olfactory sulcus—the amount of variance explained by social preferences is rather small. In other words, differences in brain anatomy contribute little to the direct prediction of individual differences in social preferences (also see note 1). However, in using a standard parcellation map, some of the larger parcels might mask variations in functionality within these parcels. A more meticulous parcellation of such areas could reveal additional relationships between cortical thickness and social preferences that were not identified here. Future work could investigate more fine-grained parcellations of different brain areas.

While people chronically differ in their social preferences, pro-social behavior often depends on and changes as a function of the environment or the history of social interactions. Such situation dependence is highly adaptive, since unconditional pro-sociality can be easily exploited and this may explain why we found no strong relations to brain anatomy. Yet pro-sociality also renders decision-making more complex. Decision-makers have to incorporate the welfare of others’ in their decision-making, and this computational complexity may manifest in brain anatomy, in particular that of the OFC.

## Supplementary Material

nsab074_Supp

## Data Availability

As the data for this study were collected in collaboration with several research groups, raw data sharing must be coordinated between co-owners and is available upon request. Derived data supporting the findings of this study will be openly available.

## Footnotes

1 Previous studies have shown some correlations between social values and gray matter volume in subcortical structures, including the amygdala (Bickart *et al.*, 2011). We explored neuroanatomical variability in subcortical nuclei by regressions social value orientation (angle, *W*_self_ and *W*_other_) on the gray matter volume of the amygdala, the caudate, the pallidum, putamen and accumbens, while controlling for age, gender and intracranial volume. Even without correcting for multiple comparisons, we found, however, no significant differences. The corresponding author can be contacted for further detail.

## Appendix

**Table A1.** Regression coefficients of social preferences on cortical thickness of brain parcels (ID, per Figure 1 and Table 1), controlling for age, gender and average cortical thickness (standard error in parentheses)

	Left hemisphere			Right hemisphere		
Parcel ID	SVO	Ws	Wo	SVO	Ws	Wo
1	1.61 e-4 (0.0004)	−0.14 (0.12)	−0.01 (0.07)	−0.749 e-4 (0.0005)	0.25 (0.13)	0.05 (0.07)
2	−4.5 e-4 (0.0003)	−0.10 (0.10)	−0.10 (0.06)	−9.50 e-4† (0.0003)	0.04 (0.10)	−0.14† (0.05)
3	−6.23 e-4 (0.0003)	0.01 (0.09)	−0.10 (0.05)	−8.27 e-4† (0.0004)	−0.03 (0.11)	−0.15† (0.06)
4	1.77 e-4 (0.0005)	0.08 (0.14)	0.03 (0.08)	2.90 e-4 (0.0004)	−0.15 (0.12)	0.01 (0.07)
5	2.43 e-4 (0.0004)	−0.12 (0.10)	0.02 (0.06)	4.29 e-4 (0.0003)	−0.12 (0.10)	0.05 (0.05)
6	1.95 e-4 (0.0003)	0.0004 (0.10)	0.02 (0.05)	2.53 e-4 (0.0003)	−0.05 (0.09)	0.02 (0.05)
7	1.23 e-4 (0.0003)	−0.08 (0.09)	−0.01 (0.05)	−3.38 e-4 (0.0004)	0.0008 (0.11)	−0.06 (0.06)
8	−3.98 e-4 (0.0005)	0.18 (0.06)	−0.01 (0.09)	4.55 e-4 (0.0006)	0.19 (0.16)	0.15 (0.09)
9	3.10 e-4 (0.0003)	0.07 (0.09)	0.06 (0.05)	4.45 e-4 (0.0003)	0.02 (0.10)	0.09 (0.05)
10	0.41 e-4 (0.0002)	−0.01 (0.06)	0.01 (0.03)	3.60 e-4 (0.0002)	−0.04 (0.07)	0.05 (0.04)
11	0.51 e-4 (0.0003)	0.03 (0.08)	0.01 (0.05)	−0.199 e-4 (0.0003)	0.02 (0.08)	0.01 (0.04)
12	3.55 e-4 (0.0003)	0.05 (0.10)	0.05 (0.06)	−6.91 e-4 (0.0003)	0.12† (0.10)	−0.10 (0.06)
13	−2.68 e-4 (0.0003)	−0.04 (0.09)	−0.05 (0.05)	1.47 e-4 (0.0003)	0.02 (0.14)	0.02 (0.05)
14	5.10 e-4 (0.0004)	0.14 (0.12)	0.08 (0.07)	4.84 e-4 (0.0005)	0.02 (0.32)	0.05 (0.08)
15	−13.0 e-4 (0.001)	0.08 (0.34)	−0.14 (0.19)	3.11 e-4 (0.0011)	−0.51 (0.32)	−0.07 (0.18)
16	−1.87 e-4 (0.0003)	−0.02 (0.07)	−0.05 (0.04)	−3.40 e-4 (0.0002)	−0.05 (0.07)	−0.06 (0.04)
17	−3.66 e-4 (0.0005)	−0.01 (0.14)	−0.05 (0.08)	6.01 e-4 (0.0005)	−0.004 (0.15)	0.13 (0.08)
18	−3.08 e-4 (0.0002)	−0.04 (0.08)	−0.05 (0.04)	1.63 e-4 (0.0003)	−0.03 (0.08)	0.03 (0.04)
19	3.79 e-4 (0.0004)	−0.11 (0.10)	0.04 (0.06)	0.758 e-4 (0.0003)	0.04 (0.08)	0.02 (0.05)
20	−1.27 e-4 (0.0002)	−0.01 (0.06)	−0.01 (0.04)	3.30 e-4 (0.0002)	−0.01 (0.07)	0.06 (0.04)
21	1.94 e-4 (0.0007)	0.0014 (0.20)	0.02 (0.11)	−8.70 e-4 (0.0006)	0.23 (0.17)	−0.09 (0.10)
22	**1.69 e-4**** (0.0005)	−0.08 (0.15)	**0.26** * (0.08)	8.77 e-4† (0.0004)	−0.08 (0.12)	0.13 (0.07)
23	3.95 e-4 (0.0004)	0.08 (0.12)	0.07 (0.07)	−2.30 e-4 (0.0004)	0.03 (0.11)	−0.03 (0.06)
24	−5.33 e-4 (0.0008)	−0.24 (0.22)	−0.15 (0.12)	−1.28 e-4 (0.0007)	−0.17 (0.20)	−0.06 (0.11)
25	−2.71 e-4 (0.0003)	−0.09 (0.09)	−0.05 (0.05)	0.281 e-4 (0.0003)	0.01 (0.09)	0.02 (0.05)
26	1.37 e-4 (0.0003)	0.12 (0.09)	0.06 (0.05)	−0.240 e-4 (0.0003)	0.24† (0.09)	0.05 (0.05)
27	−12.8 e-4 (0.0008)	−0.26 (0.24)	−0.24 (0.14)	2.21 e-4 (0.0011)	−0.23 (0.31)	−0.03 (0.18)
28	1.82 e-4 (0.0003)	−0.03 (0.08)	0.02 (0.04)	3.39 e-4 (0.0003)	−0.05 (0.07)	0.05 (0.04)
29	70.4 e-4 (0.0003)	0.06 (0.09)	0.01 (0.05)	3.62 e-4 (0.0004)	−0.17 (0.11)	−0.004 (0.06)
30	−9.25 e-4 (0.0008)	0.13 (0.22)	−0.12 (0.13)	4.11 e-4 (0.0008)	0.20 (0.22)	0.13 (0.12)
31	1.86 e-4 (0.0006)	−0.03 (0.17)	−0.02 (0.10)	1.18 e-4 (0.0007)	0.08 (0.21)	0.16 (0.12)
32	2.18 e-4 (0.0005)	−0.01 (0.16)	0.02 (0.09)	3.46 e-4 (0.0005)	0.14 (0.14)	0.07 (0.08)
33	3.22 e-4 (0.0003)	−0.06 (0.09)	0.02 (0.05)	−1.67 e-4 (0.0003)	0.03 (0.08)	−0.04 (0.04)
34	−0.031 e-4 (0.0003)	−0.09 (0.08)	−0.03 (0.05)	1.22 e-4 (0.0003)	−0.10 (0.08)	−0.01 (0.05)
35	0.448 e-4 (0.0003)	0.04 (0.08)	0.01 (0.04)	−0.457 e-4 (0.0003)	0.005 (0.09)	0.0006 (0.05)
36	−1.69 e-4 (0.0003)	−0.05 (0.09)	−0.04 (0.05)	1.58 e-4 (0.0004)	−0.08 (0.10)	0.01 (0.06)
37	−4.56 e-4 (0.0003)	0.09 (0.08)	−0.07 (0.04)	−3.36 e-4 (0.0003)	−0.02 (0.07)	−0.06 (0.04)
38	5.17 e-4 (0.0003)	−0.01 (0.09)	0.06 (0.05)	−1.78 e-4 (0.0003)	0.06 (0.08)	−0.01 (0.04)
39	1.28 e-4 (0.0005)	−0.09 (0.13)	0.02 (0.07)	6.17 e-4 (0.0005)	−0.17 (0.14)	0.07 (0.08)
40	16.3 e-4† (0.0008)	−0.27 (0.23)	0.18 (0.13)	1.60 e-4 (0.0005)	0.06 (0.14)	0.05 (0.08)
41	0.785 e-4 (0.0002)	−0.01 (0.06)	0.01 (0.04)	−2.12 e-4 (0.0002)	0.03 (0.07)	−0.02 (0.04)
42	1.50 e-4 (0.0003)	0.10 (0.10)	0.04 (0.06)	−2.38 e-4 (0.0003)	0.12 (0.09)	−0.01 (0.05)
43	2.83 e-4 (0.0002)	−0.06 (0.06)	0.02 (0.04)	−2.47 e-4 (0.0003)	−0.06 (0.08)	−0.04 (0.04)
44	1.75 e-4 (0.0003)	0.05 (0.09)	0.04 (0.05)	−1.93 e-4 (0.0003)	0.11 (0.09)	−0.002 (0.05)
45	−4.09 e-4 (0.0005)	0.03 (0.15)	−0.06 (0.09)	−5.46 e-4 (0.0005)	−0.12 (0.14)	−0.11 (0.08)
46	−4.83 e-4 (0.0004)	−0.02 (0.11)	−0.07 (0.06)	1.23 e-4 (0.0004)	−0.11 (0.10)	0.001 (0.06)
47	9.07 e-4 (0.0007)	−0.10 (0.20)	0.14 (0.11)	11.8 e-4 (0.0006)	−0.23 (0.19)	0.13 (0.10)
48	−3.05 e-4 (0.0004)	0.03 (0.11)	−0.05 (0.06)	−6.21 e-4 (0.0004)	−0.14 (0.12)	−0.13 (0.07)
49	0.788 e-4 (0.0003)	−0.16 (0.10)	−0.01 (0.05)	2.39 e-4 (0.0003)	−0.10 (0.10)	0.02 (0.05)
50	0.353 e-4 (0.0003)	0.11 (0.09)	0.03 (0.05)	−0.582 e-4 (0.0003)	0.01 (0.10)	−0.01 (0.06)
51	3.52 e-4 (0.0007)	−0.17 (0.21)	0.01 (0.12)	2.66 e-4 (0.0005)	−0.15 (0.14)	0.03 (0.08)
52	−2.28 e-4 (0.0006)	−0.18 (0.18)	−0.06 (0.10)	−1.76 e-4 (0.0008)	0.18 (0.22)	0.02 (0.12)
53	3.47 e-4 (0.0003)	−0.05 (0.09)	0.06 (0.05)	4.29 e-4 (0.0003)	0.01 (0.08)	0.07 (0.05)
54	3.75 e-4 (0.0005)	0.08 (0.14)	0.10 (0.08)	2.07 e-4 (0.0005)	0.07 (0.14)	0.06 (0.08)
55	5.08 e-4 (0.0003)	−0.05 (0.08)	0.07 (0.05)	6.16 e-4† (0.0003)	0.03 (0.09)	0.09 (0.05)
56	6.34 e-4 (0.0006)	0.10 (0.17)	0.13 (0.10)	9.06 e-4 (0.0006)	0.10 (0.16)	0.17 (0.09)
57	−5.56 e-4 (0.0005)	0.07 (0.14)	−0.08 (0.08)	−11.4 e-4† (0.0005)	0.27 (0.15)	−0.13 (0.08)
58	−2.38 e-4 (0.0004)	0.09 (0.11)	0.01 (0.06)	4.92 e-4 (0.0004)	−0.30† (0.12)	0.05 (0.07)
59	1.30 e-4 (0.0003)	−0.04 (0.09)	0.02 (0.05)	0.612 e-4 (0.0003)	−0.10 (0.10)	0.0004 (0.06)
60	3.34 e-4 (0.0003)	−0.10 (0.10)	0.05 (0.05)	4.54 e-4 (0.0004)	0.11 (0.10)	0.11 (0.06)
61	−1.15 e-4 (0.0002)	0.06 (0.06)	0.01 (0.03)	−0.854 e-4 (0.0002)	−0.02 (0.06)	−0.02 (0.03)
62	−8.47 e-4 (0.0007)	0.27 (0.19)	−0.08 (0.11)	5.76 e-4 (0.0006)	−0.20 (0.17)	0.05 (0.10)
63	−5.69 e-4 (0.0003)	−0.13 (0.10)	−0.12† (0.06)	−2.56 e-4 (0.0004)	−0.05 (0.11)	−0.04 (0.06)
64	−3.09 e-4 (0.0003)	−0.17 (0.10)	−0.08 (0.06)	−4.71 e-4 (0.0003)	0.20† (0.09)	−0.04 (0.05)
65	−2.24 e-4 (0.0003)	0.07 (0.08)	−0.03 (0.05)	−3.84 e-4 (0.0003)	0.04 (0.07)	−0.05 (0.04)
66	−1.73 e-4 (0.0004)	0.02 (0.11)	−0.03 (0.06)	−3.59 e-4 (0.0004)	0.003 (0.10)	−0.07 (0.06)
67	1.74 e-4 (0.0003)	−0.03 (0.09)	0.02 (0.05)	2.93 e-4 (0.0003)	0.09 (0.09)	0.07 (0.05)
68	1.23 e-4 (0.0003)	−0.01 (0.09)	0.02 (0.05)	0.360 e-4 (0.0003)	−0.13 (0.09)	−0.02 (0.05)
69	8.93 e-4 (0.0006)	−0.04 (0.17)	0.14 (0.10)	−2.56 e-4 (0.0006)	−0.11 (0.17)	−0.07 (0.09)
70	−6.58 e-4 (0.0004)	0.01 (0.10)	−0.11 (0.06)	−2.90 e-4 (0.0003)	−0.05 (0.08)	−0.07 (0.05)
71	2.77 e-4 (0.0003)	0.07 (0.10)	0.07 (0.05)	2.89 e-4 (0.0003)	−0.10 (0.10)	0.01 (0.06)
72	−2.77 e-4 (0.0003)	0.16 (0.10)	−0.01 (0.05)	−2.28 e-4 (0.0004)	0.22 (0.11)	0.02 (0.06)
73	−3.53 e-4 (0.0003)	0.14 (0.09)	0.00 (0.05)	−3.21 e-4 (0.0003)	−0.02 (0.09)	−0.06 (0.05)
74	2.64 e-4 (0.0004)	−0.08 (0.11)	0.03 (0.06)	−1.18 e-4 (0.0004)	−0.03 (0.10)	−0.01 (0.06)

SVO = social value orientation degree; Ws = Weight to Self; Wo = Weight to Other.

† p < 0.05 before permutations;

** p < 0.01 after permutations;

* p < 0.1 after permutations.
