# Supplementary material for: Social preferences correlate with cortical thickness of the orbito-frontal cortex
Source: Soc Cogn Affect Neurosci. 2021 Jul 9;16(11):1191–203. doi: 10.1093/scan/nsab074 (PMC8599202; doi:10.1093/scan/nsab074)
Supplement: nsab074_Supp [file nsab074_supp.zip › appendix.docx]

|  | **Left Hemisphere** | | | **Right Hemisphere** | | |
| --- | --- | --- | --- | --- | --- | --- |
| **Parcel ID** | **SVO** | **Ws** | **Wo** | **SVO** | **Ws** | **Wo** |
| 1 | 1.61 e-4 (0.0004) | -0.14  (0.12) | -0.01  (0.07) | -0.749 e-4  (0.0005) | 0.25  (0.13) | 0.05  (0.07) |
| 2 | -4.5 e-4  (0.0003) | -0.10  (0.10) | -0.10  (0.06) | -9.50 e-4†  (0.0003) | 0.04  (0.10) | -0.14†  (0.05) |
| 3 | -6.23 e-4  (0.0003) | 0.01  (0.09) | -0.10  (0.05) | -8.27 e-4†  (0.0004) | -0.03  (0.11) | -0.15†  (0.06) |
| 4 | 1.77 e-4  (0.0005) | 0.08  (0.14) | 0.03  (0.08) | 2.90 e-4  (0.0004) | -0.15  (0.12) | 0.01  (0.07) |
| 5 | 2.43 e-4  (0.0004) | -0.12  (0.10) | 0.02  (0.06) | 4.29 e-4  (0.0003) | -0.12  (0.10) | 0.05  (0.05) |
| 6 | 1.95 e-4  (0.0003) | 0.0004  (0.10) | 0.02  (0.05) | 2.53 e-4  (0.0003) | -0.05  (0.09) | 0.02  (0.05) |
| 7 | 1.23 e-4  (0.0003) | -0.08  (0.09) | -0.01  (0.05) | -3.38 e-4  (0.0004) | 0.0008  (0.11) | -0.06  (0.06) |
| 8 | -3.98 e-4  (0.0005) | 0.18  (0.06) | -0.01  (0.09) | 4.55 e-4  (0.0006) | 0.19  (0.16) | 0.15  (0.09) |
| 9 | 3.10 e-4  (0.0003) | 0.07  (0.09) | 0.06  (0.05) | 4.45 e-4  (0.0003) | 0.02  (0.10) | 0.09  (0.05) |
| 10 | 0.41 e-4  (0.0002) | -0.01  (0.06) | 0.01  (0.03) | 3.60 e-4  (0.0002) | -0.04  (0.07) | 0.05  (0.04) |
| 11 | 0.51 e-4  (0.0003) | 0.03  (0.08) | 0.01  (0.05) | -0.199 e-4  (0.0003) | 0.02  (0.08) | 0.01  (0.04) |
| 12 | 3.55 e-4  (0.0003) | 0.05  (0.10) | 0.05  (0.06) | -6.91 e-4  (0.0003) | 0.12†  (0.10) | -0.10  (0.06) |
| 13 | -2.68 e-4  (0.0003) | -0.04  (0.09) | -0.05  (0.05) | 1.47 e-4  (0.0003) | 0.02  (0.14) | 0.02  (0.05) |
| 14 | 5.10 e-4  (0.0004) | 0.14  (0.12) | 0.08  (0.07) | 4.84 e-4  (0.0005) | 0.02  (0.32) | 0.05  (0.08) |
| 15 | -13.0 e-4  (0.001) | 0.08  (0.34) | -0.14  (0.19) | 3.11 e-4  (0.0011) | -0.51  (0.32) | -0.07  (0.18) |
| 16 | -1.87 e-4  (0.0003) | -0.02  (0.07) | -0.05  (0.04) | -3.40 e-4  (0.0002) | -0.05  (0.07) | -0.06  (0.04) |
| 17 | -3.66 e-4  (0.0005) | -0.01  (0.14) | -0.05  (0.08) | 6.01 e-4  (0.0005) | -0.004  (0.15) | 0.13  (0.08) |
| 18 | -3.08 e-4  (0.0002) | -0.04  (0.08) | -0.05  (0.04) | 1.63 e-4  (0.0003) | -0.03  (0.08) | 0.03  (0.04) |
| 19 | 3.79 e-4  (0.0004) | -0.11  (0.10) | 0.04  (0.06) | 0.758 e-4  (0.0003) | 0.04  (0.08) | 0.02  (0.05) |
| 20 | -1.27 e-4  (0.0002) | -0.01  (0.06) | -0.01  (0.04) | 3.30 e-4  (0.0002) | -0.01  (0.07) | 0.06  (0.04) |
| 21 | 1.94 e-4  (0.0007) | 0.0014  (0.20) | 0.02  (0.11) | -8.70 e-4  (0.0006) | 0.23  (0.17) | -0.09  (0.10) |
| 22 | **1.69 e-4****  (0.0005) | -0.08  (0.15) | **0.26***  (0.08) | 8.77 e-4†  (0.0004) | -0.08  (0.12) | 0.13  (0.07) |
| 23 | 3.95 e-4  (0.0004) | 0.08  (0.12) | 0.07  (0.07) | -2.30 e-4  (0.0004) | 0.03  (0.11) | -0.03  (0.06) |
| 24 | -5.33 e-4  (0.0008) | -0.24  (0.22) | -0.15  (0.12) | -1.28 e-4  (0.0007) | -0.17  (0.20) | -0.06  (0.11) |
| 25 | -2.71 e-4  (0.0003) | -0.09  (0.09) | -0.05  (0.05) | 0.281 e-4  (0.0003) | 0.01  (0.09) | 0.02  (0.05) |
| 26 | 1.37 e-4  (0.0003) | 0.12  (0.09) | 0.06  (0.05) | -0.240 e-4  (0.0003) | 0.24†  (0.09) | 0.05  (0.05) |
| 27 | -12.8 e-4  (0.0008) | -0.26  (0.24) | -0.24  (0.14) | 2.21 e-4  (0.0011) | -0.23  (0.31) | -0.03  (0.18) |
| 28 | 1.82 e-4  (0.0003) | -0.03  (0.08) | 0.02  (0.04) | 3.39 e-4  (0.0003) | -0.05  (0.07) | 0.05  (0.04) |
| 29 | 70.4 e-4  (0.0003) | 0.06  (0.09) | 0.01  (0.05) | 3.62 e-4  (0.0004) | -0.17  (0.11) | -0.004  (0.06) |
| 30 | -9.25 e-4  (0.0008) | 0.13  (0.22) | -0.12  (0.13) | 4.11 e-4  (0.0008) | 0.20  (0.22) | 0.13  (0.12) |
| 31 | 1.86 e-4  (0.0006) | -0.03  (0.17) | -0.02  (0.10) | 1.18 e-4  (0.0007) | 0.08  (0.21) | 0.16  (0.12) |
| 32 | 2.18 e-4  (0.0005) | -0.01  (0.16) | 0.02  (0.09) | 3.46 e-4  (0.0005) | 0.14  (0.14) | 0.07  (0.08) |
| 33 | 3.22 e-4  (0.0003) | -0.06  (0.09) | 0.02  (0.05) | -1.67 e-4  (0.0003) | 0.03  (0.08) | -0.04  (0.04) |
| 34 | -0.031 e-4  (0.0003) | -0.09  (0.08) | -0.03  (0.05) | 1.22 e-4  (0.0003) | -0.10  (0.08) | -0.01  (0.05) |
| 35 | 0.448 e-4  (0.0003) | 0.04  (0.08) | 0.01  (0.04) | -0.457 e-4  (0.0003) | 0.005  (0.09) | 0.0006  (0.05) |
| 36 | -1.69 e-4  (0.0003) | -0.05  (0.09) | -0.04  (0.05) | 1.58 e-4  (0.0004) | -0.08  (0.10) | 0.01  (0.06) |
| 37 | -4.56 e-4  (0.0003) | 0.09  (0.08) | -0.07  (0.04) | -3.36 e-4  (0.0003) | -0.02  (0.07) | -0.06  (0.04) |
| 38 | 5.17 e-4  (0.0003) | -0.01  (0.09) | 0.06  (0.05) | -1.78 e-4  (0.0003) | 0.06  (0.08) | -0.01  (0.04) |
| 39 | 1.28 e-4  (0.0005) | -0.09  (0.13) | 0.02  (0.07) | 6.17 e-4  (0.0005) | -0.17  (0.14) | 0.07  (0.08) |
| 40 | 16.3 e-4†  (0.0008) | -0.27  (0.23) | 0.18  (0.13) | 1.60 e-4  (0.0005) | 0.06  (0.14) | 0.05  (0.08) |
| 41 | 0.785 e-4  (0.0002) | -0.01  (0.06) | 0.01  (0.04) | -2.12 e-4  (0.0002) | 0.03  (0.07) | -0.02  (0.04) |
| 42 | 1.50 e-4  (0.0003) | 0.10  (0.10) | 0.04  (0.06) | -2.38 e-4  (0.0003) | 0.12  (0.09) | -0.01  (0.05) |
| 43 | 2.83 e-4  (0.0002) | -0.06  (0.06) | 0.02  (0.04) | -2.47 e-4  (0.0003) | -0.06  (0.08) | -0.04  (0.04) |
| 44 | 1.75 e-4  (0.0003) | 0.05  (0.09) | 0.04  (0.05) | -1.93 e-4  (0.0003) | 0.11  (0.09) | -0.002  (0.05) |
| 45 | -4.09 e-4  (0.0005) | 0.03  (0.15) | -0.06  (0.09) | -5.46 e-4  (0.0005) | -0.12  (0.14) | -0.11  (0.08) |
| 46 | -4.83 e-4  (0.0004) | -0.02  (0.11) | -0.07  (0.06) | 1.23 e-4  (0.0004) | -0.11  (0.10) | 0.001  (0.06) |
| 47 | 9.07 e-4  (0.0007) | -0.10  (0.20) | 0.14  (0.11) | 11.8 e-4  (0.0006) | -0.23  (0.19) | 0.13  (0.10) |
| 48 | -3.05 e-4  (0.0004) | 0.03  (0.11) | -0.05  (0.06) | -6.21 e-4  (0.0004) | -0.14  (0.12) | -0.13  (0.07) |
| 49 | 0.788 e-4  (0.0003) | -0.16  (0.10) | -0.01  (0.05) | 2.39 e-4  (0.0003) | -0.10  (0.10) | 0.02  (0.05) |
| 50 | 0.353 e-4  (0.0003) | 0.11  (0.09) | 0.03  (0.05) | -0.582 e-4  (0.0003) | 0.01  (0.10) | -0.01  (0.06) |
| 51 | 3.52 e-4  (0.0007) | -0.17  (0.21) | 0.01  (0.12) | 2.66 e-4  (0.0005) | -0.15  (0.14) | 0.03  (0.08) |
| 52 | -2.28 e-4  (0.0006) | -0.18  (0.18) | -0.06  (0.10) | -1.76 e-4  (0.0008) | 0.18  (0.22) | 0.02  (0.12) |
| 53 | 3.47 e-4  (0.0003) | -0.05  (0.09) | 0.06  (0.05) | 4.29 e-4  (0.0003) | 0.01  (0.08) | 0.07  (0.05) |
| 54 | 3.75 e-4  (0.0005) | 0.08  (0.14) | 0.10  (0.08) | 2.07 e-4  (0.0005) | 0.07  (0.14) | 0.06  (0.08) |
| 55 | 5.08 e-4  (0.0003) | -0.05  (0.08) | 0.07  (0.05) | 6.16 e-4†  (0.0003) | 0.03  (0.09) | 0.09  (0.05) |
| 56 | 6.34 e-4  (0.0006) | 0.10  (0.17) | 0.13  (0.10) | 9.06 e-4  (0.0006) | 0.10  (0.16) | 0.17  (0.09) |
| 57 | -5.56 e-4  (0.0005) | 0.07  (0.14) | -0.08  (0.08) | -11.4 e-4†  (0.0005) | 0.27  (0.15) | -0.13  (0.08) |
| 58 | -2.38 e-4  (0.0004) | 0.09  (0.11) | 0.01  (0.06) | 4.92 e-4  (0.0004) | -0.30†  (0.12) | 0.05  (0.07) |
| 59 | 1.30 e-4  (0.0003) | -0.04  (0.09) | 0.02  (0.05) | 0.612 e-4  (0.0003) | -0.10  (0.10) | 0.0004  (0.06) |
| 60 | 3.34 e-4  (0.0003) | -0.10  (0.10) | 0.05  (0.05) | 4.54 e-4  (0.0004) | 0.11  (0.10) | 0.11  (0.06) |
| 61 | -1.15 e-4  (0.0002) | 0.06  (0.06) | 0.01  (0.03) | -0.854 e-4  (0.0002) | -0.02  (0.06) | -0.02  (0.03) |
| 62 | -8.47 e-4  (0.0007) | 0.27  (0.19) | -0.08  (0.11) | 5.76 e-4  (0.0006) | -0.20  (0.17) | 0.05  (0.10) |
| 63 | -5.69 e-4  (0.0003) | -0.13  (0.10) | -0.12†  (0.06) | -2.56 e-4  (0.0004) | -0.05  (0.11) | -0.04  (0.06) |
| 64 | -3.09 e-4  (0.0003) | -0.17  (0.10) | -0.08  (0.06) | -4.71 e-4  (0.0003) | 0.20†  (0.09) | -0.04  (0.05) |
| 65 | -2.24 e-4  (0.0003) | 0.07  (0.08) | -0.03  (0.05) | -3.84 e-4  (0.0003) | 0.04  (0.07) | -0.05  (0.04) |
| 66 | -1.73 e-4  (0.0004) | 0.02  (0.11) | -0.03  (0.06) | -3.59 e-4  (0.0004) | 0.003  (0.10) | -0.07  (0.06) |
| 67 | 1.74 e-4  (0.0003) | -0.03  (0.09) | 0.02  (0.05) | 2.93 e-4  (0.0003) | 0.09  (0.09) | 0.07  (0.05) |
| 68 | 1.23 e-4  (0.0003) | -0.01  (0.09) | 0.02  (0.05) | 0.360 e-4  (0.0003) | -0.13  (0.09) | -0.02  (0.05) |
| 69 | 8.93 e-4  (0.0006) | -0.04  (0.17) | 0.14  (0.10) | -2.56 e-4  (0.0006) | -0.11  (0.17) | -0.07  (0.09) |
| 70 | -6.58 e-4  (0.0004) | 0.01  (0.10) | -0.11  (0.06) | -2.90 e-4  (0.0003) | -0.05  (0.08) | -0.07  (0.05) |
| 71 | 2.77 e-4  (0.0003) | 0.07  (0.10) | 0.07  (0.05) | 2.89 e-4  (0.0003) | -0.10  (0.10) | 0.01  (0.06) |
| 72 | -2.77 e-4  (0.0003) | 0.16  (0.10) | -0.01  (0.05) | -2.28 e-4  (0.0004) | 0.22  (0.11) | 0.02  (0.06) |
| 73 | -3.53 e-4  (0.0003) | 0.14  (0.09) | 0.00  (0.05) | -3.21 e-4  (0.0003) | -0.02  (0.09) | -0.06  (0.05) |
| 74 | 2.64 e-4  (0.0004) | -0.08  (0.11) | 0.03  (0.06) | -1.18 e-4  (0.0004) | -0.03  (0.10) | -0.01  (0.06) |
