# Supplementary material for: Social preferences correlate with cortical thickness of the orbito-frontal cortex
Source: Soc Cogn Affect Neurosci. 2021 Jul 9;16(11):1191–203. doi: 10.1093/scan/nsab074 (PMC8599202; doi:10.1093/scan/nsab074)
Supplement: nsab074_Supp [file nsab074_supp.zip › Supplement.docx]

***Online Supplement***

**Table S1** Archetypal SVOs. Adapted from Murphy and Ackerman (2014).

| **Archetypal Social Value Orientations** | | | | | |
| --- | --- | --- | --- | --- | --- |
| Self | Other | Orientation | Inferred Motivation | Weight on one’s own outcome | Weight on other’s outcome |
| 25.6 | 25.6 | Prosocial | Maximize the joint payoff / minimize the difference between payoffs | 1 | 1 |
| 30 | 15 | Individualistic | Maximize the payoff to oneself | 1 | 0 |
| 25.6 | 4.4 | Competitive | Maximize the positive difference between the payoff for oneself and the payoff for the other | 1 | -1 |
| 15 | 0 | Sadistic | Minimize the other’s payoff | 0 | -1 |
| 4.4 | 4.4 | Sadomasochistic | Minimize the joint payoff or minimize the difference between payoffs | -1 | -1 |
| 0 | 15 | Masochistic | Minimize the payoff to oneself | -1 | 0 |
| 4.4 | 25.6 | Martyr | Maximize the negative difference between the other’s payoff and the payoff for oneself | -1 | 1 |
| 15 | 30 | Altruistic | Maximize the other’s payoff | 0 | 1 |

**Table S2** List of questions asked to participants to obtain a measure of their Social Value Orientation. Questions were asked in random order.

| Question |  | Self | Other |  | Question |  | Self | Other |
| --- | --- | --- | --- | --- | --- | --- | --- | --- |
| 1. | A | 15 | 30 |  | 13. | A | 15 | 0 |
|  | B | 18.9 | 29.5 |  |  | B | 11.1 | 0.5 |
| 2. | A | 18.9 | 29.5 |  | 14. | A | 11.1 | 0.5 |
|  | B | 22.5 | 28 |  |  | B | 7.5 | 2 |
| 3. | A | 22.5 | 28 |  | 15. | A | 7.5 | 2 |
|  | B | 25.6 | 25.6 |  |  | B | 4.4 | 4.4 |
| 4. | A | 25.6 | 25.6 |  | 16. | A | 4.4 | 4.4 |
|  | B | 28 | 22.5 |  |  | B | 2 | 7.5 |
| 5. | A | 28 | 22.5 |  | 17. | A | 2 | 7.5 |
|  | B | 29.5 | 18.9 |  |  | B | 0.5 | 11.1 |
| 6. | A | 29.5 | 18.9 |  | 18. | A | 0.5 | 11.1 |
|  | B | 30 | 15 |  |  | B | 0 | 15 |
| 7. | A | 30 | 15 |  | 19. | A | 0 | 15 |
|  | B | 29.5 | 11.1 |  |  | B | 0.5 | 18.9 |
| 8. | A | 29.5 | 11.1 |  | 20. | A | 0.5 | 18.9 |
|  | B | 28 | 7.5 |  |  | B | 2 | 22.5 |
| 9. | A | 28 | 7.5 |  | 21. | A | 2 | 22.5 |
|  | B | 25.6 | 4.4 |  |  | B | 4.4 | 25.6 |
| 10. | A | 25.6 | 4.4 |  | 22. | A | 4.4 | 25.6 |
|  | B | 22.5 | 2 |  |  | B | 7.5 | 28 |
| 11. | A | 22.5 | 2 |  | 23. | A | 7.5 | 28 |
|  | B | 18.9 | 0.5 |  |  | B | 11.1 | 29.5 |
| 12. | A | 18.9 | 0.5 |  | 24. | A | 11.1 | 29.5 |
|  | B | 15 | 0 |  |  | B | 15 | 30 |

--- Figure S1 here ---
