# Supplementary figures and images for: Social preferences correlate with cortical thickness of the orbito-frontal cortex
Source: Soc Cogn Affect Neurosci. 2021 Jul 9;16(11):1191–203. doi: 10.1093/scan/nsab074 (PMC8599202; doi:10.1093/scan/nsab074)

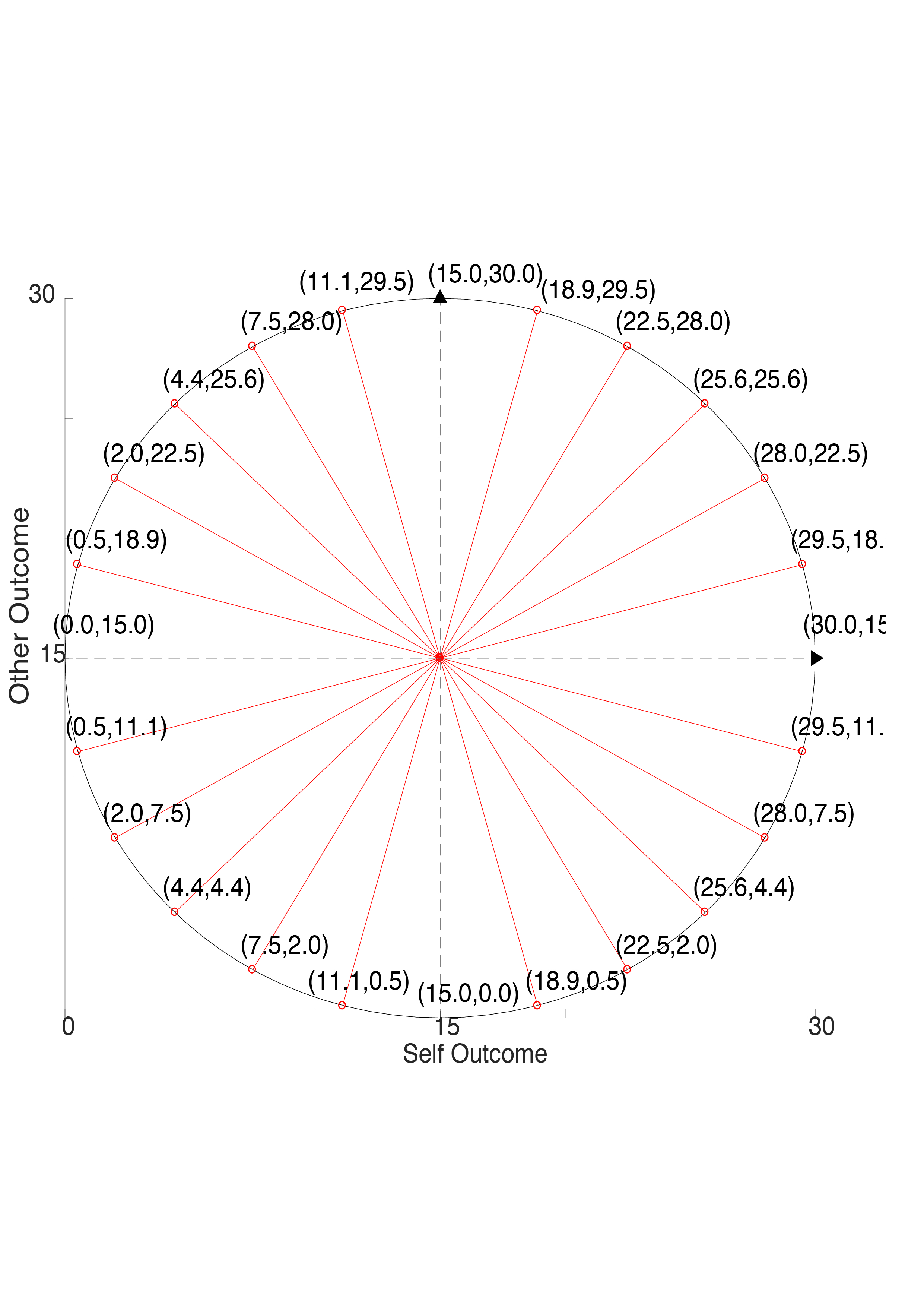

Supplement: nsab074_Supp [file nsab074_supp.zip › scan-20-284-File008.jpg]
